# Effect of sandblasting on Shear Bond Strength of 3D Printed Denture Base to conventional and Milled Denture Teeth

**DOI:** 10.1186/s12903-026-08672-1

**Published:** 2026-06-13

**Authors:** Abdelrahman I. Hleeba, Mohammed G. ElKafrawy, Fadel Elsaid Abdelfattah

**Affiliations:** Prosthodontic Department, Faculty of Dentistry, Tanta University, Tanta, Egypt

**Keywords:** 3d printed, Denture base, Milled teeth, Conventional teeth, Sandblasting, Shear bond strength

## Abstract

**Purpose:**

This study evaluated the effect of sandblasting on the shear bond strength (SBS) of three-dimensional (3D) printed denture base resin bonded to both conventional and milled artificial teeth.

**Materials and methods:**

Forty-eight specimens were divided according to the application of sandblasting to fitting surface of experimental teeth into 3 main equal groups, Group A: 16 specimens without sandblasting. Group B: 16 specimens with sandblasting using 50 μm aluminum oxide Al₂O₃, Group C: 16 specimens with sandblasting using 250 μm Al₂O₃, each main group was subdivided according to the type of artificial teeth into two equal subgroups, 8 specimens for each. All teeth were vertically aligned and bonded to a cylindrical base of three dimensional(3D) printed denture base resin. The SBS of specimen was measured at a cross-head speed of 1 mm/min until debonding. Data were statistically analyzed using two-way ANOVA, one-way ANOVA with Bonferroni post-hoc, and Student’s t-tests. Failure modes were analyzed using Chi-square and Monte Carlo tests (*P* ≤ 0.05).

**Results:**

Both sandblasting and tooth type significantly influenced SBS (*p* < 0.001), with sandblasting showing a dominant effect (Partial η² = 0.981). Milled teeth demonstrated superior bonding compared to conventional teeth across all groups (*p* < 0.001). SBS values increased proportionally with Al₂O₃, Al2O3 particle size (Group A < B < C), with the highest SBS recorded for milled teeth in Group C (1.514 ± 0.029 MPa). Furthermore, a significant shift in failure modes was observed (*p* < 0.001).

**Conclusion:**

Within the limitations of this in-vitro study, it can be concluded that increasing sandblasting intensity significantly enhances the bond strength of denture bases. 3D printed denture base with milled teeth provide significantly better interfacial integrity and higher SBS compared to conventional acrylic teeth.

## Introduction

Polymethyl methacrylate (PMMA) remains a prevalent choice for fabricating denture bases. Its widespread clinical application is attributed to several advantageous characteristics, such as excellent biocompatibility within the oral environment and a lack of toxicity [1, 2]. Furthermore, it exhibits significant dimensional precision, is neutral in taste, and provides reliable bonding to prosthetic teeth [3]. 

For the prosthetic restoration of individuals without natural teeth, acrylic-based resins are standard materials for manufacturing artificial teeth in removable appliances [4, 5]. When selecting these components, the physical and mechanical characteristics are critical factors for clinical success. Currently, the market offers a diverse range of options, varying from traditional acrylics to advanced formulations such as high cross-linked resins and composite-based teeth [6].

With the advancement of digital technology in recent years, computer-aided design and computer-assisted manufacturing (CAD/CAM) and 3D printing have been widely applied in dentistry [7–9]. Denture teeth are milled from pre-polymerized acrylic resin blocks that were formed under intense heat and pressure.

Modern CAD/CAM techniques for producing complete dentures primarily utilize either the milling of pre-polymerized resin discs or additive 3D printing with light-curing resins. Once the denture teeth and base are fabricated, they are integrated through a specialized proprietary bonding system [10]. 

The three-dimensional (3D) printed denture uses additive technology to create a denture following digital design, 3D-printed denture enable more efficient clinical adaptation which minimizes patient discomfort and potentially reduces long-term residual bone resorption [11, 12].

Bonding between the denture base material and the artificial teeth is imperative for the completeness of dentures and the patient’s quality of life. Understanding the types of bonds involved in bonding artificial teeth to denture bases is crucial for optimizing dental prosthetic fabrication techniques. Various bonding techniques are utilized in dentistry, each with distinct characteristics impacting bond strength and durability [13].

In digital workflows, such as CAD/CAM (Computer-Aided Design/Computer-Aided Manufacturing), teeth can be separately fabricated through 3D printing or milling processes before being bonded to the printed denture base. This approach allows for precise customization and alignment of teeth, improving aesthetics and potentially reducing prosthesis failure [11].

Debonding of a tooth from a denture base of a complete or partial removable denture is the most common clinical situation requiring subsequent repair [14]. Studies have shown that bond failure at the tooth-denture base interface accounts for roughly 22% to 30% of all denture repairs performed by commercial dental laboratories [15, 16].

Bonding integrity between acrylic teeth and the denture base can be influenced by several factors, including occlusion, the alignment of teeth relative to the alveolar ridge, and the ridge-lap surface area (RLSA). Furthermore, the specific types of denture base materials, processing techniques, and modifications made to the acrylic teeth all play a role in potential interface failure [17]. 

Sandblasting is a mechanical method commonly used for treating surfaces, where a high-pressure aluminum oxide spray is used to treat the ridge-lap surface of the denture teeth [18]. This mechanism induces roughening, irregularity, and porosity of ridge-lap surface. It can facilitate mechanical interlocking that promotes the bonding between the denture tooth and the denture base [18, 19]. Aluminum oxide of various particle sizes has been employed to enhance the bond between the acrylic teeth and the denture base resin [20–22].

Shear bond strength testing is the most widely used type of testing [23] for analyzing the bond strength between denture base resins and artificial teeth [24, 25]. 

However, despite the versatility of sandblasting as a surface pretreatment for prosthetic teeth, there is currently a lack of consensus regarding the optimal particle size for aluminum oxide Al₂O₃ when bonding to 3D-printed denture bases. Most existing research focuses on the behavior of teeth bonded to conventional PMMA, whereas the interaction between various sandblasted tooth surfaces and the unique chemistry of 3D-printed resins remains insufficiently explored.

This scientific uncertainty regarding the ideal abrasive intensity creates a research gap in understanding how particle size directly dictates the micromechanical interlocking and clinical longevity of the tooth-base interface. Therefore, a strong rationale exists for evaluating different Al₂O₃ particle sizes on different tooth types to identify the most effective surface activation method and reduce the high incidence of debonding failures observed in digital dentures [22].

Two null hypotheses were addressed in the present study. The first hypothesis stated that there would be no significant difference in the shear bond strength of experimental denture teeth bonded to a 3D-printed denture base regarding the two different sizes of aluminum oxide sandblasting. Similarly, the second null hypothesis proposed that no significant difference exists in the shear bond strength to the 3D-printed denture base among the two different types of denture teeth investigated.

## Materials and methods

### The materials and devices used in this study are summarized in Table 1

**Fig. 1:**
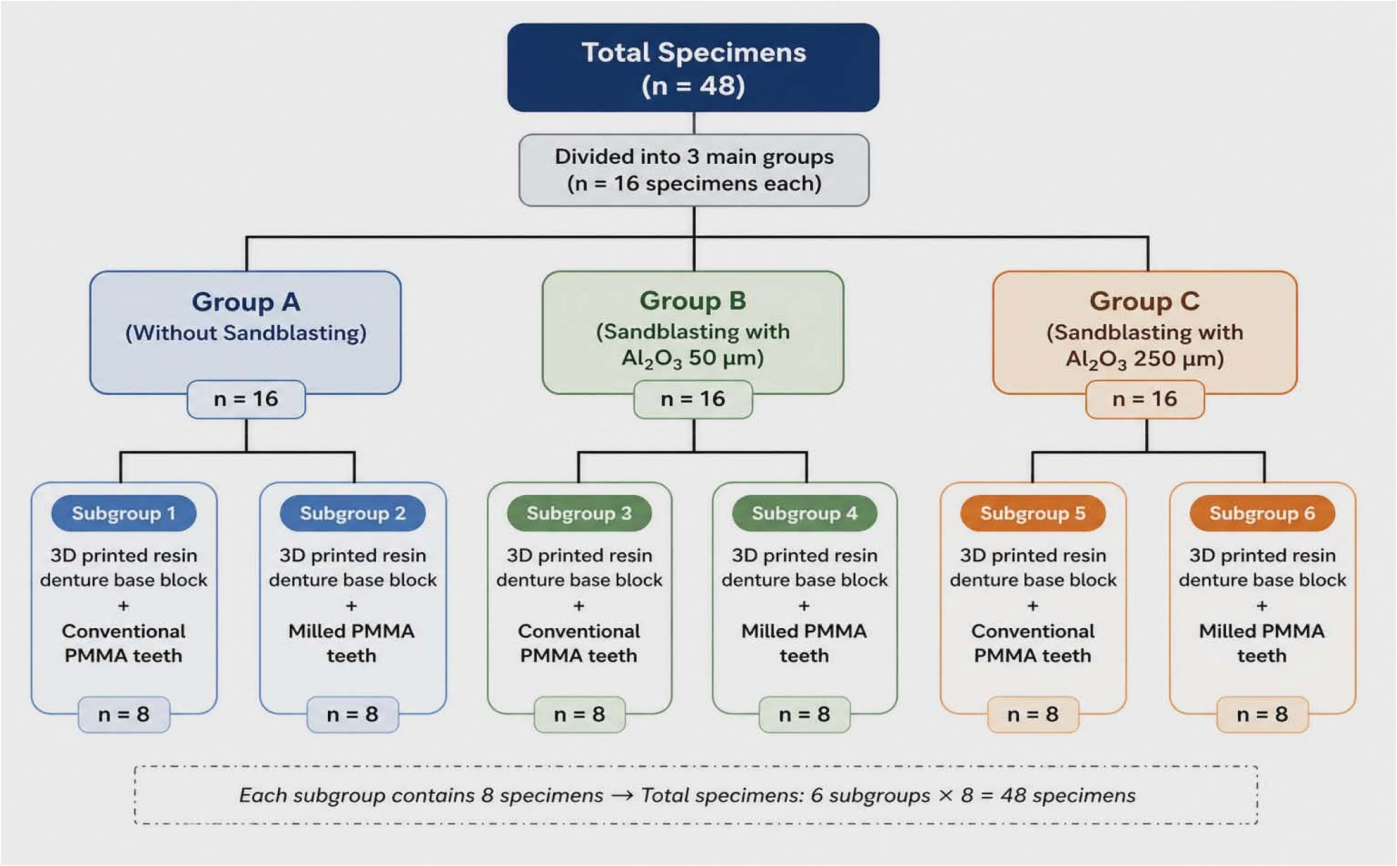
Eexperimental study groups

**Fig. 2:**
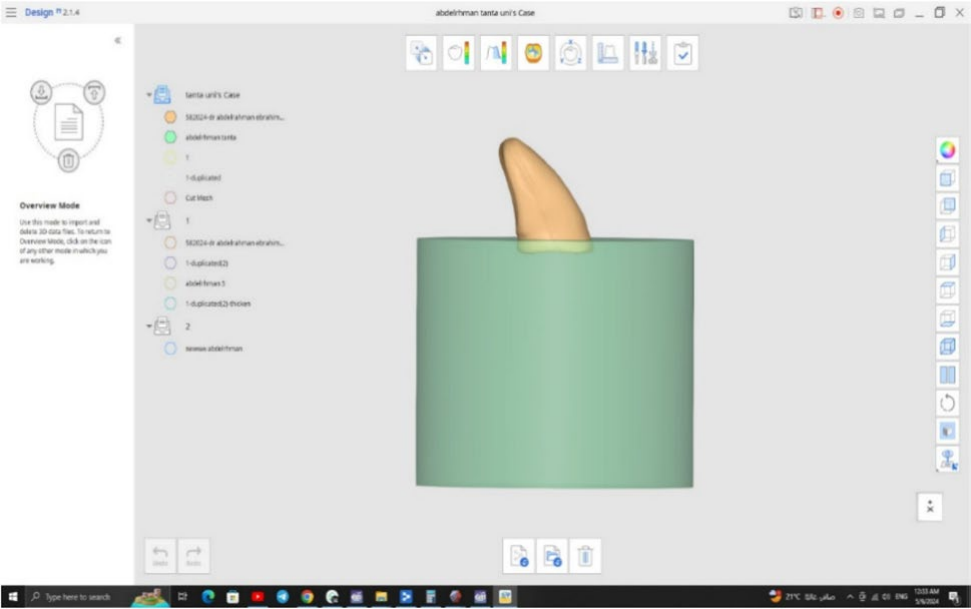
Integration of the STL file of the experimental tooth into the virtual design of the denture base block to create a socket for tooth placement

A total of forty-eight specimens were divided into three main equal groups (*n* = 16) based on the sandblasting treatment applied to the fitting surface: Group A (no sandblasting), Group B (sandblasting with 50 μm Al₂O₃), and Group C (sandblasting with 250 μm Al₂O₃). Each main group was further subdivided into two equal subgroups (*n* = 8) according to the type of artificial teeth used, as illustrated in Fig. 1. All specimens were fabricated in accordance with the International Organization for Standardization (ISO) standards (ISO/TS 19736) [26]. The design of each specimen consisted of a left maxillary central incisor vertically aligned within a cylindrical base (height = 20 mm, diameter = 25 mm), extending 1 mm above the neck of the tooth, as shown in Fig. 2

**Table 1:** Material and devices used in this study

Material	Manufacture
Unpolymerized resin (3D printed resin) Ultra Resin (x-denture)	Ege Dental Endüstri, Turkey
PMMA disc	Upcera, China
Conventional acrylic teeth	Eraylar Akrilik A.Ş., Turkey
Bonding agent (Visio. Link)	Bredent GmbH, Germany
Composite resin (combo. lign)	Bredent GmbH, Germany
Phrozen sonic mighty 4k (3D printer machine)	Phrozen Technology, Taiwan
ROLAND DWX-52DCI (milling machine)	Roland DG Corporation, Japan
Medit I 1700 (Intra oral scanner)	Medit link, south Korea
Sand blasting machine (Renfert)	Renfert, Germany
Universal testing machine	Instron Corb, USA
Scanning electron microscope	JEOL, Japan

### Sample size calculations

The total sample size for this study, after calculating the dropout rate, was 48 specimens (subdivided into 16 specimens in each group). The sample size was determined based on El Refay et al. (2023) with an effect size = 0.5726109. The significance level was set at 0.05 and the statistical power was more than 80%. The sample size was calculated using G*Power software (version 3.1.9.7).

### Specimen fabrication

#### Milled PMMA teeth

**Fig. 3:**
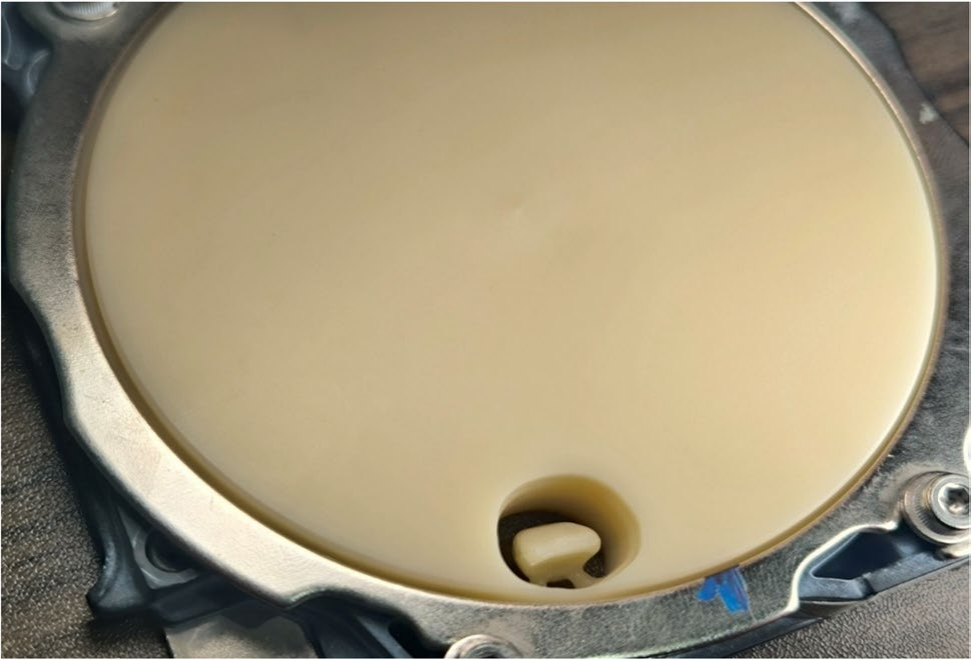
Completion of the milling process

To standardize dimensions of all experimental teeth, a conventional acrylic tooth (left maxillary central incisor) was scanned using a Medit I 1700 intra oral scanner, and the data were exported as an STL file. Subsequently, the STL file was imported into a nesting software program (MILLBOX DGSHAPE V3.7.3) and exported to the milling machine software (VPanel DWX). The milling process was then performed under dry conditions using a 5-axis Roland DWX-52DCi Dental Milling Machine, as shown in Fig. 3.

#### 3D printed denture base block

The dimensions of the denture base block (height: 20 mm, diameter: 25 mm) were designed using MEDIT design software. To create a 1 mm deep socket corresponding to the anatomy of the tooth neck, the STL file of the experimental tooth was integrated into the virtual design of the block. Subsequently, the final design was imported into Chitubox nesting software (v1.7.0) and printed using a Phrozen 3D printer at a zero-degree build angle. Post-printing, the blocks were removed, washed under running water to eliminate residual resin, and soaked in 99% isopropyl alcohol for 10 min. After thorough drying, the specimens were cured for 15 min in a light polymerization device (Bre. Lux Power Unit 2, Bredent GmbH, Senden, Germany). According to the manufacturer’s specifications and the mechanical properties evaluated, this 3D-printed resin is classified as a definitive (permanent) restorative material.

#### Sandblasting by Al₂O₃ to fitting surface of experimental teeth

The fitting surfaces of the teeth were treated using a sand blasting machine of two particle sizes of aluminum oxide 50 μm and 250 μm respectively. Using a Renfert sandblasting machine**).** for 10 s at a distance 1 cm from the fitting teeth surface. To ensure standardized distance, a custom-made holding fixture (jig) was utilized. The nozzle of the sandblasting unit was rigidly secured to the jig at a fixed distance of 10 mm from the specimen surface, maintaining a constant 90-degree inclination angle for all samples throughout the treatment. 

#### Cementation methods

**Fig. 4:**
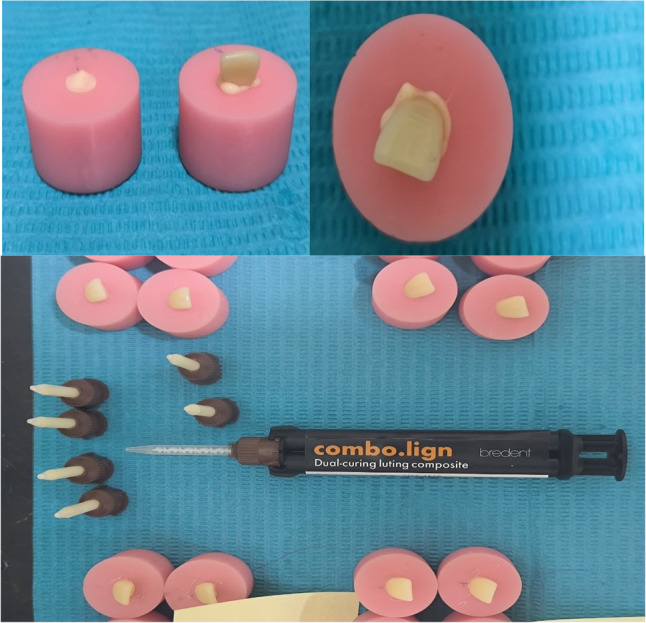
Cementation of experimental teeth into the denture base block sockets

The cementation of conventional and milled teeth to the 3D-printed denture base was performed by applying a thin, uniform layer of Visio.link (Bredent GmbH, Senden, Germany). The adhesive was applied to the fitting surface of all teeth and the socket area of the 3D-printed denture base block. Both the teeth and the 3D-printed denture base blocks were then cured in a light polymerization device (Bre. Lux Power Unit 2, Bredent GmbH, Senden, Germany) for 90 s. Subsequently, an amount of luting composite (combo. lign, Bredent GmbH, Senden, Germany) was applied into the denture socket until completely filled. The denture teeth were then seated within the sockets of the denture base, and any excess composite cement was carefully removed to prevent any dislodgment of teeth. Finally, the assembly was cured in a light polymerization device for 180 seconds [27], as shown in Fig. 4. 

#### Shear bond strength testing method

**Fig. 5:**
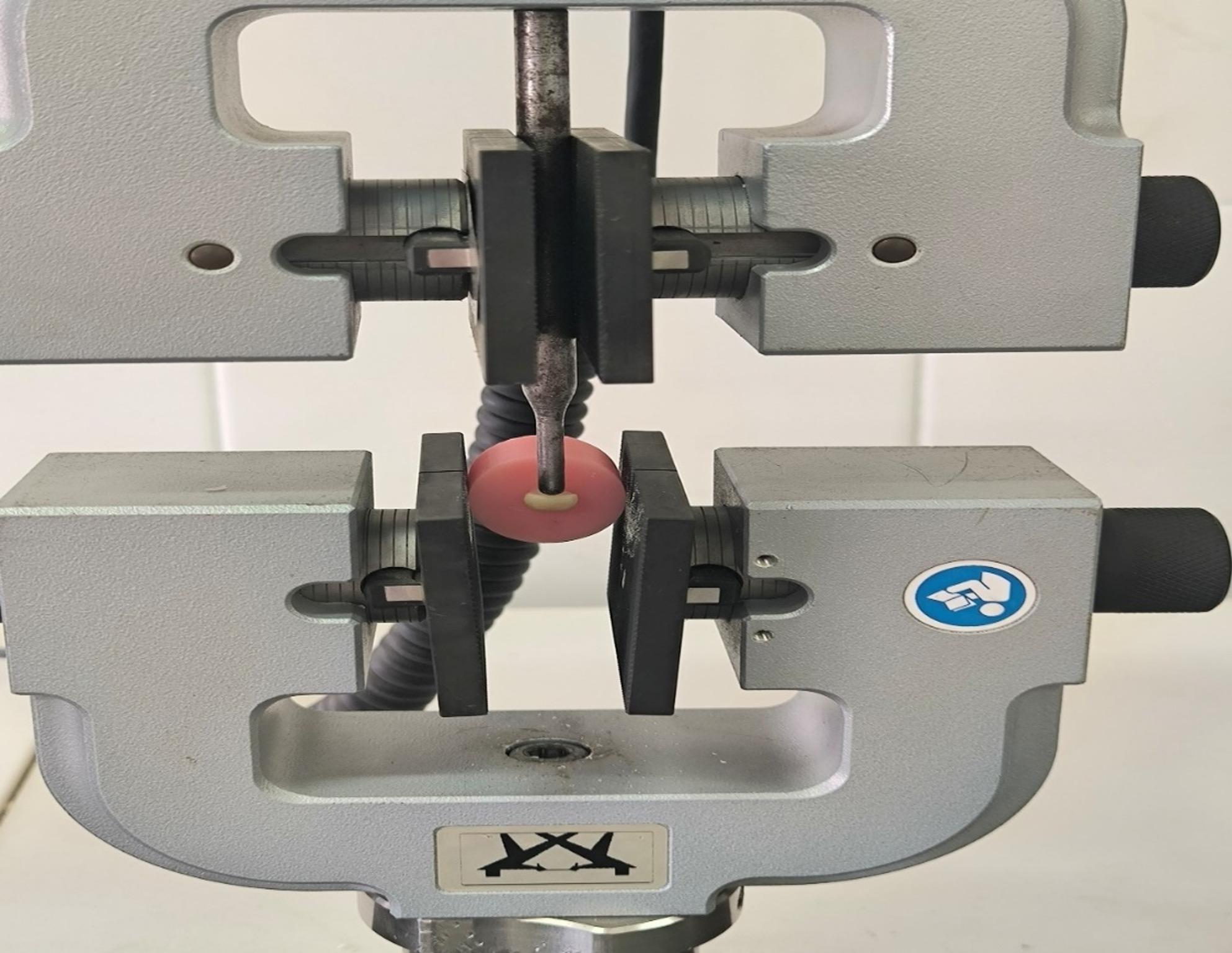
Specimens subjected to the shear bond strength test

A universal testing machine (Instron Model 3365; Instron Corb, Canton, MA, USA) with a cylindrical flat shear pin was used for the application of the load, the load direction was directed on the palatal surface of the tooth, vertical (approximately. 90°) to the long axis of the tooth. The shear pin shall be with half of its area contact and apply the load on the incisal edge of the tooth with cross head speed of 1 mm/min until the specimen fracture was occurred. The failure load was recorded at fracture, the fractured surfaces were carefully inspected and stored for further evaluation [26], as illustrated in Fig. 5.

Shear bond strength was calculated via the following formula: BS = F/A, where (F) represent maximum load at fracture and (A) denotes bonding surface area, which was determined to be 40.866 mm^2^.

The surface area (40.866 mm^2^) was calculated digitally by scanning the internal fitting surface of the tooth and utilizing the measurement tools within the Medit Design software suite to extract the precise bonding interface area. 

#### Failure mode determination

**Fig. 6:**
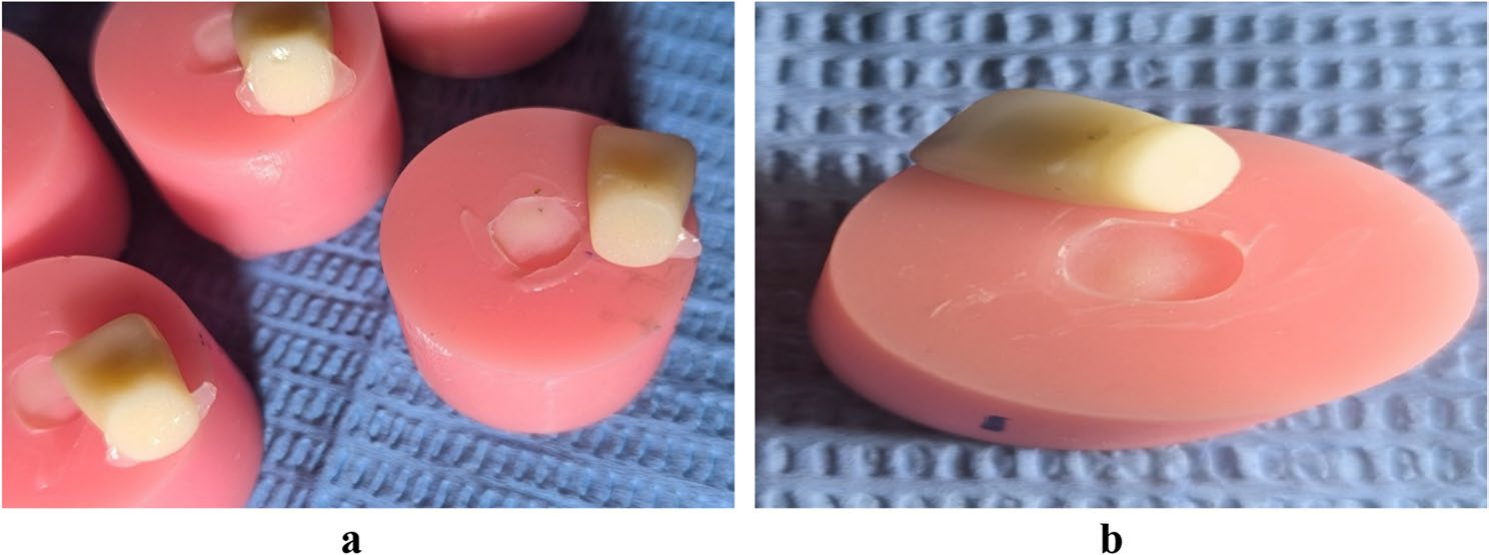
**a** Specimens after fracture, demonstrating a mixed failure mode. **b** Specimens after fracture, exhibiting an adhesive failure mode

All fractured specimens were examined under a scanning electron microscope (SEM) to determine the mode of fracture. As per ISO/TS 19,736 standards, a fracture path along the interface between the tooth and the base was described as an adhesive fracture. Cohesive fracture involves a fracture path either within the tooth or within the base. If the fracture path was partially adhesive and partially cohesive, it was described as a mixed fracture, as shown in Fig. 6a, b.

### Statistical analyses

#### Statistical analysis of the data

Data were entered into the computer and analyzed using the IBM SPSS software package version 27.0. (Armonk, NY: IBM Corp, released in 2020). Categorical data were represented as numbers and percentages. A Chi-square test was applied to compare between different groups. When more than 20% of the cells had an expected count less than 5, the Monte Carlo correction test was applied for tables larger than 2 × 2. For continuous data, normality was tested using the Shapiro-Wilk test. Quantitative data were expressed as ranges (minimum and maximum), means, standard deviations, and median. For normally distributed quantitative variables, shear bond strength (MPa) was expressed as mean ± standard deviation (SD). A two-way ANOVA analysis was performed to evaluate the effects of sandblasting (three levels), type of teeth (two levels), and their interaction. When a statistically significant main effect or interaction was detected, pairwise comparisons were carried out using one-way ANOVA to compare the different sandblasting groups within each type of teeth, followed by Bonferroni-adjusted multiple comparisons in addition to independent samples Student’s t-test was applied to compare between conventional and milled PMMA teeth within each sandblasting group. The Significance of the obtained results was judged at the 5% level and all tests were two-tailed. Effect sizes were reported using partial eta squared (partial η²) for ANOVA.

## Result

Table 2 demonstrates that both sandblasting and the type of teeth have a highly statistically significant effect on shear bond strength (*p* < 0.001 for both). The effect of sandblasting is particularly dominant, with an exceptionally large effect size (Partial η² = 0.981), indicating that it accounts for nearly all variability in bond strength. Similarly, the type of teeth also shows a strong influence (η² = 0.888).

Moreover, the interaction between sandblasting and the type of teeth is statistically significant (*p* = 0.025), suggesting that the effect of sandblasting differs depending on the type of teeth. However, the interaction effect size is relatively small (η² = 0.161) compared to the main effects, indicating that the combined influence is less substantial than that of each factor individually.

**Table 2:** Two-way ANOVA comparing the effects of sandblasting and type of teeth on Shear bond strength (Mpa)

Source	Type III Sum of squares	df	Mean Square	F-value	*p*-value	Partial η²
Sandblasting	1.745	2	0.872	1079.986	< 0.001^*^	0.981
Type of teeth	0.268	1	0.268	331.812	< 0.001^*^	0.888
Sandblasting ⋅ Type of teeth	0.007	2	0.003	4.024	0.025^*^	0.161

Levene's Test of Equality of Error Variances: F=1.525; p=0.203

Partial η²: Partial Eta Square

*: Statistically significant at p ≤ 0.05 (All tests were two-tailed)

Table 3 reveals a consistent and statistically significant increase in shear bond strength across the three sandblasting conditions (Group A → B → C) for both conventional and milled PMMA teeth (*p* < 0.001). Post hoc analysis confirms that all pairwise comparisons between groups are statistically significant, as indicated by different superscript letters.

Additionally, milled PMMA teeth exhibited significantly higher bond strength than conventional PMMA teeth under all sandblasting conditions (*p* < 0.001), highlighting the superiority of milled teeth in bonding performance. The very small standard deviations indicate minimal variability within groups, contributing to the highly significant results. Overall, the findings suggest that increasing sandblasting intensity enhances bond strength, with the highest values observed in the aluminum oxide 250 μm treatment, particularly for milled PMMA teeth.

**Table 3:** Comparison between the different studied groups according to shear bond strength (Mpa)

Sand blasting Type of teeth	Group A	Group B	Group C	F-value	*P*-value
Conventional PMMA teeth	0.890^c^ ± 0.035	1.163^b^ ± 0.022	1.381^a^ ± 0.034	504.099^*^	< 0.001^*^
Milled PMMA Teeth	1.072^c^ ± 0.017	1.296^b^ ± 0.029	1.514^a^ ± 0.029	597.793^*^	< 0.001^*^
T	13.227^*^	10.474^*^	8.336^*^		
p_0_	< 0.001^*^	< 0.001^*^	< 0.001^*^		

8 replicas for each group Data were expressed using Mean ± Standard deviation (SD.), Means in the same Row with any Small Common letter (a-c) are not significant (OR means with totally different letters (a-c) are significant)

Group A: No sand blasting

Group B: Sandblasting 50 μm

Group C: Sand blasting aluminum oxide 250 μm

t: Student t-test F: F for One way ANOVA test, pairwise comparison bet. each 2 groups were done using Post Hoc Test (adjusted Bonferroni

p: p value for comparing between the three studied Sand blasting in each Type of teeth

p_0_: p value for comparing between Conventional and Milled in each Sand blasting

*: Statistically significant at *p* ≤ 0.05 (All tests were two-tailed)

Table 4 demonstrates a highly statistically significant difference in the distribution of fracture modes across the studied groups (χ² = 27.732, MC *p* < 0.001), indicating that both the sandblasting condition and the type of teeth significantly influence the mode of fracture.

**Fig. 7:**
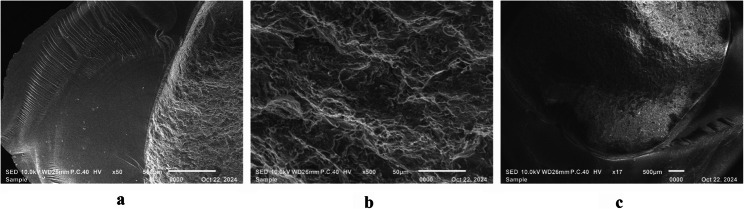
**a** SEM image of specimen after fracture. demonstrating a mixed failure. **b** SEM image of the tooth surface after fracture. **c** SEM image of the polished surface of denture base block after fracture

In Group A (no sandblasting), all conventional PMMA samples (100%) exhibited adhesive fracture, whereas milled PMMA showed a mixed pattern, with 25% adhesive and 75% mixed fractures. This suggests relatively weak interfacial bonding in the absence of surface treatment, particularly for conventional teeth. With the introduction of sandblasting (Group B, 50 μm), there was a marked shift toward mixed fracture in both types of teeth. Conventional PMMA showed 87.5% mixed fractures, while milled PMMA reached 100% mixed fractures. This transition indicates a significant improvement in bond strength and interfacial integrity. In Group C (250 μm aluminum oxide), all samples (100%)—both conventional and milled—demonstrated mixed fracture exclusively, with the complete disappearance of adhesive failure. This pattern strongly reflects enhanced bonding performance due to increased surface roughness and micromechanical retention. The use of superscript letters (a–b) confirms that: Group A (conventional) is significantly different from all other subgroups. No significant differences are observed among the remaining groups, as they share common letters, indicating a convergence toward similar fracture behavior under sandblasting conditions. Notably, cohesive fracture was absent in all groups, suggesting that failure consistently occurred at or near the interface rather than within the bulk material, even under enhanced bonding conditions. The mode of fracture is shown in Fig. 7a, b, and c. illustrating the orientation of the fracture lines within the specimens.

**Table 4:** Comparison between the different studied groups according to mode of fracture

	Group A		Group B		Group C		χ^2^	^MC^ *p*
	Conventional (*n* = 8)	Milled (*n* = 8)	Conventional (*n* = 8)	Milled (*n* = 8)	Conventional (*n* = 8)	Milled (*n* = 8)		
Mode of fracture								
Adhesive fracture	8^a^ (100.0%)	2^b^ (25.0%)	1^b^ (12.5%)	0^b^ (0.0%)	0^b^ (0.0%)	0^b^ (0.0%)	27.732^*^	< 0.001^*^
Mixed fracture	0^a^ (0.0%)	6^b^ (75.0%)	7^b^ (87.5%)	8^b^ (100.0%)	8^b^ (100.0%)	8^b^ (100.0%)		
Cohesive fracture	0 (0.0%)	0 (0.0%)	0 (0.0%)	0 (0.0%)	0 (0.0%)	0 (0.0%)		

Group A: No sand blasting

Group B: Sandblasting 50 μm

Group C: Sand blasting aluminum oxide 250 μm

χ^2^: Chi square test MC: Monte Carlo test

p: p value for comparing between the studied groups

*: Statistically significant at *p* ≤ 0.05 (All tests were two-tailed)

Frequency with any Small Common letter ^(a−b)^ is not significant (OR Frequency with totally different letters ^(a−b)^ are significant)

## Discussion

The bonding integrity between denture teeth and the 3D-printed base is a multifactorial phenomenon governed by the interplay of surface energy, mechanical interlocking, and substrate density. The current study evaluated the effect of sandblasting on the shear bond strength (SBS) between a 3D-printed denture base bonded to two types of acrylic denture teeth.

Overall, surface treatment via sandblasting significantly improved the bond strength compared to the untreated group. The statistical dominance of this treatment was confirmed by Two-way ANOVA, which revealed an exceptionally large effect size (Partial η² = 0.981), indicating that mechanical modification is the primary factor in bond optimization. The results showed a consistent and statistically significant increase in SBS across the sandblasting conditions from Group A to Group C. This improvement is in agreement with El-Refay [21] and Bahrani et al. [28], who demonstrated that sandblasting improves bond strength compared to non-sandblasted surfaces, and Mahadevan et al. [29], who found that sandblasting the ridge-lap area significantly enhances SBS. Mechanistically, this can be explained by the fact that Al2O3 particles remove surface contaminants and the glazed “lube” layer, creating a roughened topographic pattern that increases the surface energy and creates extensive micromechanical interlocking sites. Specifically, the highest SBS was observed in the 250 μm Al2O3 treated group (Group C), followed by the 50 μm group. This finding is consistent with Consani et al. [30], who reported greater values for larger particle sizes, and Ghea et al. [22], who found that 250 μm particles significantly enhance the bond between acrylic teeth and thermoplastic bases. The explanatory reason for this superiority is the higher kinetic energy of larger particles, which creates deeper micro-pits on the ridge-lap surface, providing a more robust foundation for the bonding agent.

In the current study, milled PMMA teeth recorded significantly higher SBS values compared to conventional PMMA teeth in all specimens. These results align with Prpić et al. [31] and Mohamed et al. [10], who both found that CAD/CAM milled materials generally provide better bonding characteristics than conventional heat-cured alternatives. Similarly, El Harbi et al. [32] showed that bond strength to digital materials can be optimized to exceed conventional setups. Scientifically, the superiority of milled teeth is attributed to their industrial manufacturing process, which involves high-pressure polymerization, resulting in a highly dense, cross-linked structure with fewer internal voids. When sandblasted, this dense matrix maintains a more stable and well-defined retentive pattern compared to the relatively less converted conventional PMMA. However, our findings disagreed with Choi et al. [33], which may be due to differences in the specific 3D-printing parameters or the type of bonding agents utilized.

The mode of failure was described as adhesive for untreated conventional subgroups and mixed for all other treated groups. A possible reason for the adhesive failure is that no modification was applied to the glazing ridge-lap surface of the conventional teeth, leaving it too smooth for effective bonding, a finding consistent with El Harbi et al. [32] and Mohamed et al. [10]. The shift toward a mixed failure pattern in the sandblasted groups, especially in Group C (100% mixed failure), indicates that the bond strength was high enough to distribute stresses into the material itself. While El-Refay [21] found 100% adhesive failure in all groups, our results showed a transition to mixed failure, possibly due to differences in the bonding agents or the design of the denture base used in this study. Consequently, based on these findings, the first null hypothesis of this study was rejected as sandblasting significantly improved the shear bond strength of all specimens. Similarly, the second null hypothesis was also rejected, as the results demonstrated that the shear bond strength of the 3D-printed denture base bonded to milled teeth was significantly better than when bonded to conventional teeth.

## Conclusion

This study demonstrates that while increasing alumina particle size significantly enhances the shear bond strength of PMMA teeth, the effect is highly dependent on the manufacturing method of the substrate. Milled CAD/CAM PMMA exhibits a superior bonding capacity and a more favorable fracture pattern compared to conventional PMMA under identical sandblasting conditions. The transition from adhesive to predominantly mixed failure modes in the milled groups—even at moderate sandblasting intensities—underscores the enhanced surface reactivity of digital materials. Ultimately, these findings indicate that the effectiveness of surface conditioning is closely linked to the material’s microstructure. For CAD/CAM restorations, the use of larger particle sizes (250 μm) appears to offer a more reliable pathway to achieving a stable interfacial bond and a predictable fracture pattern, providing a practical clinical approach to enhancing the overall durability of the prosthetic assembly.

## Limitation

### Single resin material

This study evaluated only one type of 3D-printed denture base resin. Due to variations in chemical composition and polymerization among different 3D-printing systems, these results may not be generalizable to all available digital denture materials. 

### Lack of artificial aging

The specimens were tested shortly after fabrication without undergoing thermocycling or long-term water storage. Consequently, the findings reflect the initial bond strength and do not account for the long-term clinical durability.

The use of shear bond strength test does not fully simulate the complex, multi-directional, and cyclic functional loads (fatigue) that occur in the oral cavity during mastication.

## Data Availability

The data supporting the findings of this study are available upon reasonable request from the corresponding author.
